# Draft Genome Sequence of *Enterobacter* sp. Strain A8, a Carbazole-Degrading Bacterium

**DOI:** 10.1128/MRA.00301-19

**Published:** 2019-05-02

**Authors:** Nidhi Gupta, Kelly A. Skinner, Samiya Khan, Janaka N. Edirisinghe, Christopher S. Henry

**Affiliations:** a Argonne National Laboratory (ANL), Argonne, Illinois, USA; b University of Chicago, Chicago, Illinois, USA; c Jaypee Institute of Information Technology, Noida, Uttar Pradesh, India; Broad Institute of MIT and Harvard University

## Abstract

We present here the draft genome sequence of a carbazole-degrading *Enterobacter* species. The draft genome sequence will provide insight into various genes involved in the degradation of carbazole and other related aromatic compounds.

## ANNOUNCEMENT

Carbazole and its derivatives are used as a feedstock in dye, plastic, and pharmaceutical industries and are naturally found in crude oil, shale oil, and creosote (1). These compounds are known to be mutagenic and carcinogenic. While prospecting for new molecular mechanisms for carbazole degradation, a carbazole-degrading bacterium, *Enterobacter* sp. strain A8, was isolated from hydrocarbon-contaminated soil (Gujarat, India) by enrichment culture, as described by Singh et al. (2). The bacterium could degrade 83% of 500 ppm (initial concentration) carbazole in 240 hours (2). *Enterobacter* sp. strains are Gram-negative Proteobacteria members belonging to the family Enterobacteriaceae. They colonize various environments and are reported to have diverse metabolic activities, heavy metal tolerances, antibiotic resistances, and biological control agent properties (3–5). Having the whole-genome sequence of the bacterium will help us decipher the genes involved not only in carbazole degradation but also those involved in other catabolic activities.

For genomic DNA isolation, a single colony from an LB plate was used to inoculate an overnight LB culture at 30°C with shaking at 200 rpm. A bacterial genomic DNA isolation kit (DNeasy PowerSoil kit; Qiagen) was used to extract genomic DNA from the overnight culture. The genomic DNA (1 ng) was used to create a paired-end library using the Nextera XT DNA library preparation kit by Illumina. The library was sequenced using an Illumina MiSeq system, producing 3,861,561 paired-end sequences (1.3 Gb total; average length, 251 bp). We utilized the Department of Energy’s KBase system (6) for contig generation and genome annotation (https://narrative.kbase.us/). First, the sequence was uploaded into KBase in FASTQ format, and then adaptor sequences were trimmed from both ends using Trimmomatic v0.36 with default parameters. Reads were assembled using SPAdes v3.12.0 (7) with default settings, generating a draft genome of 4.9 Mb in 21 contigs with a total GC content of 54.85%. The smallest contig in the assembly was 533 bp, and the largest contig was 1,701,541 bp, with an *N*_50_ length of 810,037 bp.

For annotation purposes, Prokka v1.12 (8) was applied with default parameters, resulting in the identification of 4,555 protein-coding genes in the *Enterobacter* sp. strain A8 genome. The genes in the genome were subsequently reannotated with Rapid Annotations using Subsystems Technology (RAST) (9), with 1,351 (30%) of the genes being assigned to SEED subsystems and with 3,617 (79%) of the genes having nonhypothetical functions. Subsystems signifying the survival of the isolate in aromatic compound-contaminated soil (gene counts) included membrane transport (75), stress response (70), metabolism of aromatic compounds (22), and motility and chemotaxis (109). The annotation analysis of *Enterobacter* sp. strain A8 did not reveal the presence of any of the protein families known to be involved in the degradation of carbazole or anthranilate (10). However, genes involved in the degradation of phenylpropionate, phenylacetate, and vanillate were annotated. Reported genes for antibiotic resistance (*acrAB*, *ampC*, *ampD*, and *ampR*) and Pythium ultimum resistance (*cyaA*) were annotated in the *Enterobacter* sp. strain A8 genome. In addition, genes involved in resistance to heavy metals, such as chromium, arsenic, zinc, nickel, cobalt, and magnesium, were also annotated in the genome.

### Data availability.

The complete genome sequence for *Enterobacter* sp. strain A8 and the raw sequence data have been deposited in GenBank (accession numbers SCMF01000001 to SCMF01000021) and the Sequence Read Archive (accession number SRS4256731).
